# Fat Mass and Obesity-Related (FTO) Gene Variant Is a Predictor of CVD in T2DM Patients

**DOI:** 10.1155/2024/5914316

**Published:** 2024-09-02

**Authors:** Mazhar Hussain, Akbar Waheed, Asim Elahi, Ghulam Mustafa

**Affiliations:** ^1^ Pharmacology Department Sheikh Zayed Medical College, Rahim Yar Khan, Punjab, Pakistan; ^2^ Pharmacology Department Riphah International University, Islamabad, Pakistan; ^3^ Internal Medicine Residency Program South Texas Health GME Consortium Texas A & M School of Medicine, Bryan, Texas, USA; ^4^ Community Medicine Department Sheikh Zayed Medical College, Rahim Yar Khan, Punjab, Pakistan

**Keywords:** BMI, CVD, FTO gene, lipid profile, T2DM

## Abstract

**Background:** The role of the common FTO gene variant rs9939609 in obesity has been well established, and the FTO gene has a strong association with T2DM.

**Objective:** To investigate the association of FTO gene variant rs9939609 with obesity-related parameters in T2DM and CVD patients.

**Materials and Methods:** In this cross-sectional study, 280 subjects of either sex aged 45.10 ± 9.6 years were randomly divided into four groups, that is, T2DM, T2DM with CVD, nondiabetic with CVD disease, and normal control. These samples were genotyped by ARMS-PCR. The FTO gene association with obesity-related parameters in T2DM and CVD patients was analyzed by SPSS 22.

**Results:** The TT genotype was the most common genotype (46.80%) in our study groups. The minor allele frequency (MAF) was significantly higher in T2DM patients (0.39 vs. 0.28), T2DM patients with CVD (0.43 vs. 0.28), and nondiabetic patients with CVD (0.35 vs. 0.28) as compared to control with *p* < 0.005. Carriers of the AA genotype of the FTO gene rs9939609 were significantly associated with increased BMI, WC, HbA1C, SBP, DBP, and TGs and lowered HDL cholesterol as compared to the TA and TT genotypes in T2DM and CVD patients with *p* < 0.005. The FTO gene variant rs9939609 showed a significant association with T2DM and CVD. The AA genotype odds ratio (OR) in T2DM was 1.48 (1.06–2.32), *p* = 0.006, and in CVD, it was 1.56 (1.04–2.4), *p* = 0.003.

**Conclusion:** The FTO gene variant rs9939609 has a strong association with T2DM and CVD. The AA genotype of FTO gene variants rs9939609 showed a strong association with most of the risk factors of CVD and T2DM.

## 1. Introduction

T2DM is a complex metabolic disorder and a therapeutic challenge all across the world. In 2019, 463 million (9.3%) people were affected by this chronic metabolic disorder worldwide. This number is expected to reach 578 million (10.2%) by 2030 and 700 million (10.9%) by 2045. The number of cases of T2DM is projected to increase by 51% over a period of 26 years (2019–2045). It is estimated that approximately 50.1% of people are unaware of their diabetes [1]. Pakistan stands in third position in the world diabetes ranking after China and India. In 2022, 33 million (26.7%) adults were living with diabetes in Pakistan. This number will be expected to increase at an alarming rate and pose an enormous burden on the health system [2].

Patients with T2DM have a 2–4-fold increased risk of CVD morbidity than nondiabetics. The mortality risk in T2DM due to CVD is also two to six times more as compared to nondiabetics. CVD is present in 32.2% of people with T2DM, mostly in the form of ischemic heart disease, stroke, and heart failure. More than 50% of deaths in T2DM patients are due to CVD. Nearly 80% of diabetes-related deaths occur in low- and middle-income countries [3].

The FTO gene is located on Chromosome Number 16 (16 q12.2) and has nine axons and many single nucleotide polymorphisms (SNPs). These SNPs are located on Intron 1 of the FTO gene, a point of intense linkage disequilibrium. The most commonly studied SNPs are rs1421085, rs17817499, rs9939609, and rs8050136. These SNPs have a strong association with energy homeostasis, food intake, physical activity, T2DM, nonalcoholic fatty liver disease, and CVD [4]. FTO gene variants have a strong association with obesity, metabolic syndrome, inflammation, hypertension, dyslipidemia, polycystic ovarian disease, and various cancers [5].

FTO gene variants also have a strong association with T2DM. The studies on FTO gene variants rs9939609 and rs8050136 were conducted in East Asia, South Asia, and Europe, while in North America and Africa, the most studied FTO gene variants were rs1421085 and rs17817499. In a meta-analysis of various studies, the FTO gene variants rs9939609 and rs8050136 predispose to T2DM independently of BMI, while other studies concluded that the association of T2DM with these genetic variants did not depend upon BMI [6, 7].

There is a strong need to investigate the association of the FTO gene variant with T2DM and CVD patients on the basis of BMI and other conventional risk factors. Studies revealed that the FTO gene rs9939609 variant showed a strong association with CVD in the European population. This association was not dependent on any conventional risk factors of CVD. These remarkable findings need to be investigated in the Asian population too [8, 9].

In this study, we investigated the association of the FTO gene variant rs9939609 with obesity-related parameters in T2DM and CVD patients.

## 2. Material and Methods

This cross-sectional study was comprised of 280 subjects divided into four groups, with 70 in each group. The data were analyzed for 183 (65.4%) males and 97 (34.60%) females, with a mean age of 45.10 ± 9.6 years. Patients were recruited from the cardiology department and diabetic clinic of Sheikh Zayed Medical College and Hospital, Rahim Yar Khan, located in the south region of Punjab, over a period of 6 months, from January to June 2021. Group A was comprised of T2DM patients, Group B was comprised of T2DM patients with CVD, Group C was comprised of nondiabetic patients with CVD, and Group D was comprised of normal control. T2DM patients in Groups A and B were diagnosed on the basis of IDF criteria [10]. CVD patients in Groups B and C were diagnosed on the basis of ECG, cardiac catheterization, cardiac enzymes, and angiography. Patients in Group D were taken from a similar cohort and served as normal controls without diabetes and CVD. The study participants were divided into blocks of four through the block randomization technique. The study participants were allocated a specific number through computed software. Data about family history, medical history, and lifestyle habits were collected from clinical records. Information regarding physical activity and smoking status (current/past) were collected on a standard, valid questionnaire. BMI was categorized as normal, overweight, and obese according to Asian Pacific guidelines [11].

Patients with renal impairment, CNS diseases, hepatic dysfunction, endocrine disorders, pregnancy, malignancy, lactation, insulin therapy, autoimmune disease, and inflammatory disease on the basis of medical records and history were excluded from the study. Patients in Groups A and B were taking oral antidiabetic medications, while patients in Groups B and C were taking lipid-lowering drugs. A sample size of 280 subjects was calculated by using Rao Soft keeping a confidence level of 95% and a 24% minor allele frequency (MAF) of FTO SNP rs9939609 on the basis of BMI. The study was approved by the ethical committee of the institutional review board (58/IRB/SZMC/SZH), and the study perspectives were clearly explained to all participants before obtaining informed consent. The research protocols were performed according to the Declaration of Helsinki and good principle practice (GCP) guidelines.

Body weight was measured by a digital weight machine without shoes. Height was measured by microtoise. The waist circumference was determined by placing the measuring tape horizontally around the abdomen at the level of the iliac crest. BMI was calculated as weight (kilograms)/height (square meters). Blood pressure was measured in the supine position in both arms twice to avoid errors at intervals of 15 min by the mercury sphygmomanometer apparatus.

A fasting venous blood sample (10 mL) was collected by venipuncture using a disposable syringe. Five milliliters of blood was transferred into a EDTA tube for DNA extraction and HbA1C measurement. The remaining 5 mL of blood was centrifuged to collect serum for the analysis of fasting blood glucose (FBG) and lipid profile. The collection and measurement of blood samples were performed according to standard procedures and protocols. The FBG was determined via the standard glucose oxidase method using an automated analyzer. The lipid profile was analyzed by the automated enzymatic method (Hitachi, Tokyo, Japan). High-performance liquid chromatography was used to determine HbA1C.

### 2.1. Genotyping

DNA was extracted from a whole peripheral blood sample using the DNA extraction kit (Promega Wizard®). DNA samples were amplified by PCR and master mix polymerase. The FTO gene variant rs9939609 genotypes were analyzed by the tetraprimer amplification refractory mutation system polymerase chain reaction (ARMS-PCR) method.

### 2.2. Thermal Cycling Protocols

The double-stranded DNA was initially denatured at 95°C for 1 min, and the final denaturation was done at 95°C for 1 min. Annealing was done at 65°C, while extension was done at 72°C for 1 min and 30 s. The final extension was done at 72°C for 3 min. The amplified product was run on 6% polyacrylamide gels, followed by staining with silver nitrate.

### 2.3. Statistical Analysis

A statistical package for social sciences (SPSS 22) was used to analyze the data. Continuous variables were expressed as mean ± SD, while categorical variables were expressed as a percentage. Student's *t*-test was used to compare normal distribution data in two groups, while ANOVA was used for more than two groups. A chi-squared test was used to determine allele frequencies and genotype distribution between study groups. Chi-squared test, Student's *t*-test, and ANOVA were used to analyze data within groups. An ANOVA test was used to determine the effect of the FTO gene rs9939609 variant on various study parameters. The study groups were tested for the Hardy–Weinberg equilibrium. The association between the FTO gene variant with diabetes and CVD was tested by univariate logistic regression with unadjusted odds ratios (ORs) and a 95% confidence interval. *p* value < 0.05 was considered to be statistically significant.

## 3. Results

The mean age of T2DM patients with CVD and nondiabetic patients with CVD was significantly higher as compared to T2DM patients and controls (*p* = 0.001), showing the occurrence of CVD with advancing age. No significant gender difference was observed among study groups (*p* = 0.947). Higher frequencies of smoking (*p* < 0.001) were recorded in T2DM patients with CVD when compared to T2DM patients and controls. Most of these baseline parameters were significantly different as compared to the control group. These baseline characteristics are shown in Table 1.

**Figure 1:**
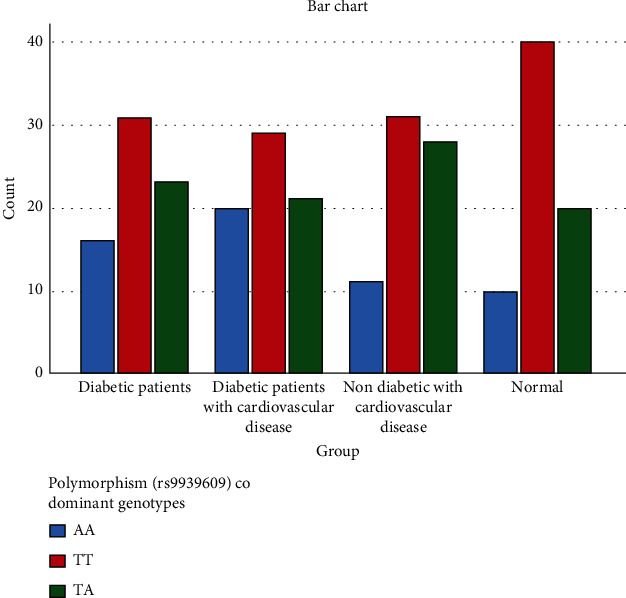
Genotype frequency of the FTO gene variant (rs9939609) in various study groups.

Allele and genotype frequencies of the FTO gene variant rs9939609 are shown in Figure 1. The allele frequencies of FTO gene variant rs9939609 were in the Hardy–Weinberg equilibrium in all study groups (*p* > 0.05). The TT genotype was the most common genotype (46.80%) in our study groups. The MAF was significantly higher in T2DM patients (0.39 vs. 0.28) as compared to the control group. Similarly, MAF was significantly higher in T2DM patients with CVD (0.43 vs. 0.28) and nondiabetic patients with CVD (0.35 vs. 0.28) as compared to control. The FTO gene variant rs9939609 was found to be significantly associated with T2DM and CVD. The AA genotype OR for T2DM was 1.48 (1.06–2.32), *p*-0.006, and for CVD, it was 1.56 (1.04–2.4) (*p* = 0.003) (Table 2).

**Figure 2:**
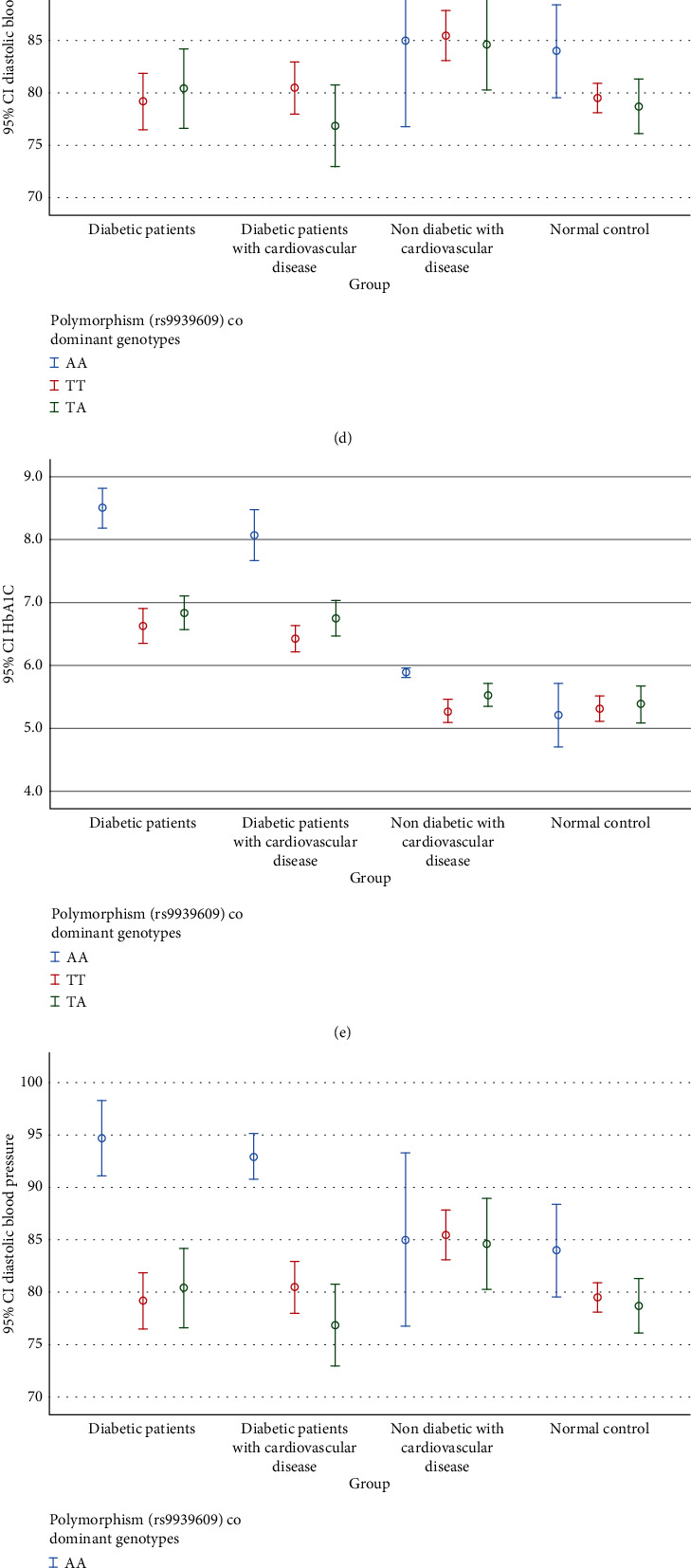
(a) Body weight (kilograms). (b) Body mass index (kilograms per square meter). (c) Systolic blood pressure (millimeters of mercury). (d) Diastolic blood pressure (millimeters of mercury). (e) HbA1C (percent). (f) Total cholesterol (milligrams per deciliter). (g) Serum triglycerides (milligrams per deciliter). (h) Low-density lipoprotein cholesterol (milligrams per deciliter). (i) High-density lipoprotein cholesterol (milligrams per deciliter).

Table 3 and Figures 2(a), 2(b), 2(c), 2(d), 2(e), 2(f), 2(g), 2(h), and 2(i) show that carriers of the risk AA genotype of the FTO variant rs9939609 had increased BMI (<0.001) versus the TT and TA genotypes in T2DM, T2DM with CVD, and nondiabetic patients with CVD. Similarly, the AA genotype was significantly associated with an increase in waist circumference (<0.001) as compared to the TT and TA genotypes in T2DM, T2DM with CVD, and nondiabetic patients with CVD. Regarding blood pressure carriers, the AA genotype was significantly associated with increased SBP (<0.001) and DBP (<0.001) as compared to the TT and TA genotypes in T2DM and T2DM with CVD. However, no remarkable difference was found among FTO genotypes in SBP and DBP between nondiabetic patients with CVD.

Regarding glycemic control, carriers of the AA genotype were significantly associated with increased fasting blood sugar as compared to the TT genotype in T2DM and T2DM with CVD. Similarly, the AA genotype was significantly associated with increased HbA1C as compared to the TT and TA genotypes in T2DM and T2DM with CVD. We did not find any significant association of antidiabetic drugs (not shown) with different FTO genotypes in T2DM patients and T2DM patients with CVD (Table 3).

Regarding lipid profile, the carrier of the AA genotype was significantly associated with increased triglycerides as compared to the TT and TA genotypes in T2DM and T2DM with CVD. Similarly, the AA genotype was significantly associated with a decrease in HDL as compared to the TT and TA genotypes in T2DM and T2DM with CVD. The AA genotype was not significantly associated with an increase in triglycerides and a decrease in HDL-C level in nondiabetic patients with CVD. The use of statins in this group seems to have abolished the delirious effects of the AA genotype on serum triglycerides and HDL-C. However, no significant association was found between the AA genotype with serum cholesterol and low-density lipoprotein cholesterol in any study groups (Table 3).

## 4. Discussion

South Asian countries comprised 20% of the world's population. South Asian countries have a higher burden of obesity, T2DM, and CVD as compared to Caucasians. CVD prevalence ranges from 5% to 10% in South Asians as compared to 1%–2% in Caucasians. South Asians develop diabetes and its related complications at an early age due to low BMI, smaller waist circumference, increased insulin resistance, and ectopic fat deposition as compared to Caucasians. Seeing this, the World Health Organization (WHO) recommended ethnicity-based BMI values to anticipate CVD and Type 2 diabetes [12].

In our study, the AA genotype of the FTO gene variant rs9939609 showed a positive correlation with T2DM and CVD. This positive correlation was associated with BMI, waist circumference, SBP, DBP, BSF, HbA1C, triglycerides, and HDL cholesterol. An early FTO gene variant was discovered as an obesity-susceptible gene in different ethnic populations. The FTO gene association with BMI and obesity risk was replicated in various studies [13]. Subsequent studies revealed that the FTO gene was associated with T2DM on the basis of BMI, while other studies did not show this association [14]. Obesity and T2DM are two of the established risk factors for cardiovascular disease. So there is a need to investigate the effect of the FTO gene variant association on cardiovascular disease on the basis of conventional and nonconventional risk factors.

In our study, the AA genotype of the FTO gene rs9939609 has a strong association with increased BMI and waist circumference. A meta-analysis of eight studies compromising 2441 cases and 1668 controls found that the FTO gene variant rs9939609 was associated with increased obesity risk, and the A allele was considered to be a risk factor for obesity susceptibility in adults [13]. Similarly, a systematic review and meta-analysis of 23 studies among different ethnicities (Asians, Caucasians, and Amerindians) suggested that the FTO gene variants showed a positive correlation with overweight and obesity in children, adolescents, and adults [15].

In our study, individuals with the AA genotype of FTO rs9939609 had increased serum triglycerides and reduced HDL levels compared to those with the TT genotype. However, no significant difference was recorded between total cholesterol and LDL cholesterol. A study conducted by Mehrdad et al. reported that the carrier of the AA genotype of the FTO gene variant rs9939609 was associated with lower HDL-C level than the TT genotype in overweight people. On the other hand, no significant associations were found with total cholesterol, LDL cholesterol, serum insulin, and adiponectin levels [16]. In line with our study, Jalili et al. also found a significant association of the FTO gene variant with HDL, while no significant differences were found between the FTO gene variant rs9939609 and total cholesterol, LDL cholesterol, and serum triglyceride level [17].

Our results of total cholesterol, LDL cholesterol, and HDL cholesterol regarding FTO rs9939609 were inconsistent with those of Franczak et al., who observed that the AA genotype was significantly associated with lower HDL-C as compared to the TT genotype. However, no significant association was detected regarding total cholesterol and LDL cholesterol, similar to our study. Moreover, we observed a significant association between the FTO gene rs9939609 variant and serum triglyceride level as compared to this study. On the basis of the above findings, it might be concluded that the AA genotype of FTO rs9939609 causes disturbances in the lipid profile that can predispose to CVD [18].

A Go-DARTS study on 4897 diabetic patients concluded that the AA genotype of the FTO gene variant rs9939609 had a remarkable association with increased BMI, atherogenic lipid profile, and insulin resistance. This resulted in an increased risk of fatal and nonfatal myocardial infarction over a mean follow-up of 3.6 years. Treatment with statin drugs may abolish this risk [19]. Similarly, patients who were taking statins in our study (T2DM with CVD) had an improved lipid profile as compared to other groups. However, we did not notice any significant impact of SGLT-2 inhibitor drugs on CVD risk factors.

Similarly, a population-based OPERA study (Oulu Project Elucidating Risk of Atherosclerosis) over a follow-up period of 19 years showed an increased risk of coronary heart disease (CHD), CVD, and mortality with the AA genotype of the FTO rs9939609. The OPERA study postulated that the AA genotype had an independent association with CVD irrespective of age, BMI, smoking status, diabetes, and lipid profile. Although the AA genotype was also associated with increased serum FBG at baseline, this was not associated with CVD mortality. However, no significant interaction was found between the FTO genotype and statin use on CVD outcome [20]. In comparison with the OPERA study, our results showed a strong association of the AA genotype of FTO rs9939609 with CVD on the basis of BMI, BSF, HbA1C, HDL, and serum triglycerides.

However, the Finnish Diabetes Prevention Study (DPS) pointed out that the AA genotype FTO rs9939609 was associated with a 2.09-fold CVD in men with an abnormal glucose metabolism over a follow-up period of 10.2 years. Our results also showed that patients with the AA genotype FTO rs9939609 had an increase in fasting blood sugar and HbA1C. This seems that the AA genotype of FTO rs9939609 was strongly associated with disturbances in glucose metabolism [21]. On the other hand, a study conducted on 1092 cases (acute coronary syndrome [ACS]) and 1191 controls declared that the FTO gene variant rs17817449 was significantly associated with ACS in Caucasian males. This association remained significant after the exclusion of age, BMI, and diabetes mellitus in contrast to our study [22]. A study comprising 4402 controls and 1743 CHD cases in Sweden found that the A allele of the FTO gene was significantly associated with CHD in case-control studies comprising 4402 controls and 1743 CHD patients. This association was not counteracted by increased physical activity [23].

A meta-analysis of 10 studies involving 19,153 patients revealed that the FTO gene variant rs9939609 showed a strong association with the risk of CVD in the European population. This association was not mediated by changes in BMI and other conventional cardiovascular risk factors [24].

The data about the association of the FTO gene with cardiovascular disease in Asia is sparse and limited. A study was conducted in Pakistan on 970 subjects (425 CHD, 295 obese, and 250 controls) to determine the association of the FTO gene rs9939609 with CHD on the basis of serum biochemical parameters. The study revealed that a risk allele of the FTO gene rs9939609 variant was significantly associated with obesity and CHD. However, only blood glucose showed an association with the A allele of the FTO gene rs9939609 variant, while the lipid profile showed no association. They assumed that fasting blood sugar may contribute to the progression of CHD by disturbing glucose metabolism [25].

A study was conducted in Pakistan on 625 individuals to analyze the genetic risk of CAD by using 21 genetic variants, although the genetic risk scores (GRSs) of 21 SNPs were significantly higher in cases than controls and showed a significant association with CAD risk. However, the risk allele frequency of only two SNPs, APOB rs1042031 and FTO rs9939609, was associated with CAD risk. GRS also showed a positive correlation with the lipid profile [26]. A study conducted by Qureshi et al. demonstrated that the FTO gene variant was strongly associated with obesity, anthropometric, and lipid parameters in obese Pakistani individuals. They postulated that the FTO gene variant may play an important role in fat deposition by disturbing lipid metabolism [27].

The main limitation of our study was the relatively small sample size. The observation needs to be replicated in a large cohort with different ethnicities in the future to obtain a broad view. We did not observe any CVD-beneficial effects of SGLT-2 inhibitors in T2DM patients. However, this will be more clear in prospective studies to determine antidiabetic beneficial effects on different FTO genotypes. We matched the various variables of the participants that can affect the size of alleles. We also consider several confounders in statistical analysis in order to get precise results.

## 5. Conclusion

Our study indicates that the FTO gene variant rs9939609 was associated with T2DM and CVD. In our study, patients with the AA genotype of the FTO gene variant rs9939609 had increased BMI, WC, SBP, DBP, BSF, HbA1C, and TGs and a lower HDL cholesterol level. Our findings suggest that the AA genotype may be a marker of obesity, glycemic control, and lipid status in T2DM patients. Our observations further support that the FTO gene rs9939609 was an important predictor of CVD in T2DM patients on the basis of these risk factors. Moreover, further genetic studies should be conducted to add information beyond CVD risk factors in T2DM patients. This will identify the risk genotype and will help in the early identification of at-risk patients.

## Figures and Tables

**Table 1:** Baseline anthropometric, clinical, and metabolic characteristics of study groups.

**Parameters**	**T2DM**	**T2DM with CVD**	**Nondiabetic with CVD**	**Normal**	**Total**	**p** ** value**
Age (years)	42.06 ± 10.9	48.44 ± 6.1	46.53 ± 6.0	43.39 ± 12.4	45.10 ± 9.6	0.001
Sex (M)	46 (65.70%)	46 (65.70%)	46 (65.70%)	45 (64.30%)	183 (65.40%)	0.947
History of smoking	21 (30%)	30 (42.8%)	36 (51.42%)	20 (28%)	107 (38.2%)	0.020
Family history of diabetes	51 (72.9%)	39 (55.70%)	47 (67.10%)	37 (52.90%)	174 (62.10%)	0.047
BW (kg)	75.87 ± 11.31	79.27 ± 12.30	78.17 ± 10.31	77.91 ± 13.29	77.81 ± 11.85	0.392
BMI (kg/m^2^)	26.02 ± 2.29	25.72 ± 2.56	26.12 ± 2.93	21.10 ± 2.77	24.74 ± 3.38	<0.001
WC (cm)	89.30 ± 8.86	87.53 ± 8.15	91.13 ± 8.56	73.73 ± 7.22	85.42 ± 10.69	<0.001
SBP (mmHg)	123.00 ± 18.79	121.86 ± 18.00	124.93 ± 14.66	113.00 ± 5.98	120.70 ± 15.83	<0.001
DBP (mmHg)	83.14 ± 9.93	83.00 ± 9.30	83.93 ± 13.43	79.93 ± 5.21	82.50 ± 9.98	0.087
Duration of diabetes (years)	5.56 ± 2.28	8.34 ± 3.15				0.001
FBG (mg/dL)	127.94 ± 31.88	114.17 ± 27.98	81.43 ± 13.06	85.20 ± 13.66	102.19 ± 30.27	<0.001
HbA1C (%)	7.12 ± 1.02	6.98 ± 0.96	5.47 ± 0.51	5.32 ± 0.64	6.22 ± 1.16	<0.001
TC (mg/dL)	166.43 ± 26.61	170.07 ± 20.42	163.77 ± 18.69	155.96 ± 30.03	164.06 ± 24.79	0.006
LDL (mg/dL)	132.94 ± 21.96	130.70 ± 13.86	139.96 ± 17.51	108.96 ± 20.99	128.14 ± 22.05	<0.001
HDL (mg/dL)	38.44 ± 4.27	36.44 ± 4.97	41.96 ± 3.75	42.69 ± 2.36	39.88 ± 4.69	<0.001
TG (mg/dL)	186.67 ± 60.04	181.41 ± 52.33	145.86 ± 19.80	101.37 ± 17.43	153.86 ± 54.02	<0.001

*Note:* Data are presented as means ± standard deviation.

Abbreviations: BMI: body mass index; BW: body weight; DBP: diastolic blood pressure; FBG: fasting blood glucose; HbA1C: glycosylated hemoglobin; HDL-C: high-density lipoprotein cholesterol; LDL-C: low-density lipoprotein cholesterol; SBP: systolic blood pressure; TC: total cholesterol; TG: triglycerides; WC: waist circumference.

**Table 2:** Genotype/allele frequency of the FTO gene variant (rs9939609) in various study groups.

**Genotype/allele**	**Frequency in groups (** **n** = 280**)**				**OR (CI),** **p** ** value**	
	**T2DM**	**T2DM with CVD**	**Nondiabetic with CVD**	**Normal control**	**T2DM**	**CVD**
AA	16	20	11	10	1.48 (1.06–2.32), 0.006^∗^	1.56 (1.04–2.4), 0.003^∗^
TT	31	29	31	40		
TA	23	21	28	20		
T—Allele	0.60	0.56	0.64	0.71		
A—Allele	0.39	0.43	0.35	0.28		
MAF (%)	39.28%	43.5%	35.8%	28.5%		

Abbreviations: CI: confidence interval; MAF: minor allele frequency; OR: odds ratio.

^*^Significant association.

**Table 3:** Comparison of various study parameters regarding the FTO gene variant rs9939609 in various study groups.

**Parameters**	**FTO (rs9939609)**					**p** ** value**	**p** ** value**	**p** ** value**
	**Groups**	**AA**	**TT**	**TA**	**Total**	**AA/TT**	**AA/TA**	**TT/TA**
BMI (kg/m^2^)	A	28.81 ± 1.72	24.68 ± 1.45	25.89 ± 1.85	26.02 ± 2.29	<0.001	<0.001	0.025
	B	28.60 ± 2.16	23.79 ± 1.17	25.64 ± 1.61	25.72 ± 2.56	<0.001	<0.001	0.001
	C	31.18 ± 1.33	23.60 ± 0.82	26.93 ± 1.44	26.12 ± 2.93	<0.001	<0.001	<0.001
	D	20.60 ± 1.58	21.49 ± 1.47	20.58 ± 4.64	21.10 ± 2.77	0.639	1.000	0.457

WC (cm)	A	99.06 ± 5.04	85.13 ± 6.73	88.13 ± 8.47	89.30 ± 8.86	<0.001	<0.001	0.274
	B	93.80 ± 8.33	83.31 ± 6.36	87.38 ± 6.41	87.53 ± 8.14	<0.001	0.012	0.112
	C	104.45 ± 5.3	84.45 ± 4.83	93.29 ± 4.70	91.13 ± 8.55	<0.001	<0.001	<0.001
	D	70.90 ± 10.5	73.08 ± 6.89	76.45 ± 5.13	73.73 ± 7.22	0.661	0.114	0.197

SBP (mmHg)	A	145.6 ± 17.0	114.8 ± 8.90	118.2 ± 17.6	123.0 ± 18.7	<0.001	<0.001	0.659
	B	143.2 ± 15.0	112.9 ± 6.7	113.8 ± 14.0	121.8 ± 18	<0.001	<0.001	0.964
	C	130.4 ± 16.5	124.0 ± 11.5	123.7 ± 16.8	124.9 ± 14.6	0.430	0.409	0.997
	D	115.5 ± 5.9	112.8 ± 6.0	112.0 ± 5.7	113.0 ± 5.9	0.432	0.291	0.854

DBP (mmHg)	A	94.6 ± 6.70	79.19 ± 7.43	80.43 ± 8.78	83.14 ± 9.93	<0.001	<0.001	0.830
	B	93.00 ± 4.70	80.52 ± 6.46	76.90 ± 8.44	83.00 ± 9.30	<0.001	<0.001	0.152
	C	85.0 ± 12.2	85.48 ± 6.37	84.6 ± 11.23	85.07 ± 9.43	0.989	0.994	0.939
	D	84.0 ± 6.15	79.50 ± 4.36	78.75 ± 5.59	79.93 ± 5.21	0.035	0.023	0.848

FBG (mg/dL)	A	152.7 ± 29.8	113.7 ± 29.5	129.8 ± 25.6	127.9 ± 31.8	<0.001	0.041	0.104
	B	136.1 ± 27	99.7 ± 19.3	113.2 ± 26.4	114.1 ± 27.9	<0.001	0.009	0.127
	C	91.45 ± 10.	77.6 ± 11.5	81.7 ± 13.7	81.4 ± 13.0	0.006	0.075	0.414
	D	84.10 ± 11.	84.6 ± 14.3	86.9 ± 13.8	85.2 ± 13.6	0.994	0.860	0.820

HbA1C (%)	A	8.50 ± 0.58	6.62 ± 0.77	6.83 ± 0.63	7.12 ± 1.02	<0.001	<0.001	0.520
	B	8.07 ± 0.86	6.41 ± 0.53	6.74 ± 0.62	6.98 ± 0.96	<0.001	<0.001	0.202
	C	5.88 ± 0.11	5.27 ± 0.52	5.53 ± 0.48	5.47 ± 0.51	0.001	0.089	0.091
	D	5.20 ± 0.70	5.32 ± 0.63	5.38 ± 0.65	5.32 ± 0.64	0.868	0.750	0.928

TC (mg/dL)	A	168.6 ± 24.8	166.2 ± 27.5	165.1 ± 27.5	166.4 ± 26.6	0.953	0.914	0.988
	B	171.3 ± 20.1	171.1 ± 21.1	167.3 ± 20.4	170.0 ± 20.4	1.000	0.808	0.794
	C	165.9 ± 19.8	162.0 ± 18.3	164.8 ± 19.1	163.7 ± 18.6	0.826	0987	0.827
	D	145.1 ± 28.3	156.5 ± 29.1	160.1 ± 32.7	155.9 ± 30.0	0.531	0.405	0.902

TG (mg/dL)	A	249.6 ± 74.8	159.1 ± 44.6	180.0 ± 27.6	186.6 ± 60.0	<0.001	<0.001	0.273
	B	241.9 ± 60.1	147.6 ± 10.0	170.3 ± 20.6	181.4 ± 52.3	<0.001	<0.001	0.065
	C	141.6 ± 13.8	143.5 ± 20.5	150.0 ± 20.7	145.8 ± 19.7	0.960	0.464	0.432
	D	92.90 ± 14.7	100.6 ± 14.8	107.0 ± 21.8	101.3 ± 17.4	0.410	0.090	0.364

LDL-C (mg/dL)	A	139.5 ± 19.3	134.1 ± 20.7	126.8 ± 24.5	132.9 ± 21.9	0.700	0.11	0.449
	B	130.0 ± 16.2	130.7 ± 15.1	131.3 ± 9.4	130.7 ± 13.8	0.983	0.950	0.987
	C	141.6 ± 16.4	134.5 ± 20.5	145.2 ± 12.2	139.9 ± 17.5	0.469	0.832	0.049
	D	110.3 ± 19.6	109.8 ± 20.5	106.5 ± 23.3	108.9 ± 20.9	0.998	0.889	0.833

HDL-C (mg/dL)	A	34.50 ± 4.13	41.23 ± 3.03	37.43 ± 3.12	38.44 ± 4.2	<0.001	0.023	<0.001
	B	30.05 ± 2.37	41.00 ± 1.77	36.24 ± 2.07	36.44 ± 4.97	<0.001	<0.001	<0.001
	C	41.55 ± 3.11	42.00 ± 3.88	42.07 ± 3.93	41.96 ± 3.75	0.938	0.920	0.997
	D	40.70 ± 1.06	42.95 ± 2.10	43.15 ± 2.85	42.69 ± 2.36	0.016	0.017	0.943

*Note:* Data are presented as means ± standard deviation.

Abbreviations: BMI: body mass index; DBP: diastolic blood pressure; FBG: fasting blood glucose; HbA1C: glycosylated hemoglobin; HDL-C: high-density lipoprotein cholesterol; LDL-C: low-density lipoprotein cholesterol; SBP: systolic blood pressure; TC: total cholesterol; TG: triglycerides; WC: waist circumference.

## Data Availability

All relevant data have been provided in the main manuscript. The current analyzed data are available from the corresponding author and can be given upon reasonable request by email.

## References

[1] Saeedi P., Petersohn I., Salpea P. (2019). Global and regional diabetes prevalence estimates for 2019 and projections for 2030 and 2045: results from the International Diabetes Federation Diabetes Atlas, 9th edition. *Diabetes Research and Clinical Practice*.

[2] Azeem S., Khan U., Liaquat A. (2022). The increasing rate of diabetes in Pakistan: a silent killer. *Annals of Medicine and Surgery*.

[3] Roman G., Pantea S. A. (2021). Cardiovascular risk/disease in type 2 diabetes mellitus. *Type 2 Diabetes: From Pathophysiology to Cyber Systems*.

[4] Huang C., Chen W., Wang X. (2023). Studies on the fat mass and obesity-associated (FTO) gene and its impact on obesity-associated diseases. *Genes & Diseases*.

[5] Kucher A. N. (2020). The FTO Gene and diseases: the role of genetic polymorphism, epigenetic modifications, and environmental factors. *Russian Journal of Genetics*.

[6] Huong P. T., Nguyen C. T., Nhung V. T. (2021). The association between FTO polymorphisms and type 2 diabetes in Asian populations: a meta-analysis. *Meta Gene*.

[7] Ikhanjal M. A., Elouarid M. A., Zouine C. (2023). FTO gene variants (rs9939609, rs8050136 and rs17817449) and type 2 diabetes mellitus risk: a meta-analysis. *Gene*.

[8] Xu Z. Y., Jing X., Xiong X. D. (2023). Emerging role and mechanism of the FTO gene in cardiovascular diseases. *Biomolecules*.

[9] Alipour M., Rostami H., Parastouei K. (2020). Association between inflammatory obesity phenotypes, FTO-rs9939609, and cardiovascular risk factors in patients with type 2 diabetes. *Journal of Research in Medical Sciences : The Official Journal of Isfahan University of Medical Sciences*.

[10] American Diabetes Association (2021). 2. Classification and diagnosis of diabetes: standards of medical care in diabetes—2021. *Diabetes Care*.

[11] Jan A., Weir C. B. (2021). *BMI classification percentile and cut off points*.

[12] Ahmad S., Fatima S. S., Rukh G., Smith C. E. (2019). Gene lifestyle interactions with relation to obesity, cardiometabolic, and cardiovascular traits among South Asians. *Frontiers in Endocrinology*.

[13] Abd Ali A. H., Shkurat T. P., Abbas A. H. (2021). Association analysis of FTO gene polymorphisms rs9939609 and obesity risk among the adults: a systematic review and meta-analysis. *Meta Gene*.

[14] Shill L. C., Alam M. R., Chowdhury A. I., Alam S. (2021). Association of FTO gene (rs9939609) with obesity and type-2 diabetes mellitus: Review from current studies. *Romanian Journal of Diabetes Nutrition and Metabolic Diseases*.

[15] Liu C., Mou S., Cai Y. (2013). FTO gene variant and risk of overweight and obesity among children and adolescents: a systematic review and meta-analysis. *PLoS One*.

[16] Mehrdad M., Doaei S., Gholamalizadeh M., Fardaei M., Fararouei M., Eftekhari M. H. (2020). Association of FTO rs9939609 polymorphism with serum leptin, insulin, adiponectin, and lipid profile in overweight adults. *Adipocytes*.

[17] Jalili V., Mokhtari Z., Rastgoo S. (2021). The association between FTO rs9939609 polymorphism and serum lipid profile in adult women. *Diabetology and Metabolic Syndrome*.

[18] Franczak A., Kolačkov K., Jawiarczyk-Przybyłowska A., Bolanowski M. (2018). Association between FTO gene polymorphisms and HDL cholesterol concentration may cause higher risk of cardiovascular disease in patients with acromegaly. *Pituitary*.

[19] Doney A. S., Dannfald J., Kimber C. H. (2009). The FTO gene is associated with an atherogenic lipid profile and myocardial infarction in patients with type 2 diabetes: a genetics of diabetes audit and research study in Tayside Scotland (Go-DARTS) study. *Circulation. Cardiovascular Genetics*.

[20] Äijälä M., Ronkainen J., Huusko T. (2015). The fat mass and obesity-associated (FTO) gene variant rs9939609 predicts long-term incidence of cardiovascular disease and related death independent of the traditional risk factors. *Annals of Medicine*.

[21] Lappalainen T., Kolehmainen M., Schwab U. S. (2011). Association of the FTO gene variant (rs9939609) with cardiovascular disease in men with abnormal glucose metabolism–the Finnish Diabetes Prevention Study. *Nutrition, Metabolism, and Cardiovascular Diseases*.

[22] Hubacek J. A., Staněk V., Gebauerová M. (2010). A FTO variant and risk of acute coronary syndrome. *Clinica Chimica Acta*.

[23] Gustavsson J., Mehlig K., Leander K. (2014). FTO genotype, physical activity, and coronary heart disease risk in Swedish men and women. *Circulation. Cardiovascular Genetics*.

[24] Liu C., Mou S., Pan C. (2013). The FTO gene rs9939609 polymorphism predicts risk of cardiovascular disease: a systematic review and meta-analysis. *PLoS One*.

[25] Shahid S. U., Rehman A., Hasnain S. (2016). Role of a common variant of fat mass and obesity associated (FTO) gene in obesity and coronary artery disease in subjects from Punjab, Pakistan: a case control study. *Lipids in Health and Disease*.

[26] Shahid S. U., Cooper J. A., Beaney K. E., Li K., Rehman A., Humphries S. E. (2017). Genetic risk analysis of coronary artery disease in Pakistani subjects using a genetic risk score of 21 variants. *Atherosclerosis*.

[27] Qureshi S. A., Mumtaz A., Shahid S. U., Shabana N. A. (2017). rs3751812, a common variant in fat mass and obesity-associated (FTO) gene, is associated with serum high-and low-density lipoprotein cholesterol in Pakistani individuals. *Nutrition*.

